# Improving Orthodontic Bracket Adhesion to Monolithic Zirconia: Pretreatment Protocols Across Different Zirconia Types

**DOI:** 10.3290/j.jad.c_2668

**Published:** 2026-05-29

**Authors:** Natalie Kuipers, Matthias Kern, Sebastian Wille, Frank Lehmann, Sinan Sen, Maximiliane Amelie Schlenz, Alexander Schmidt

**Affiliations:** a Natalie Kuipers Assistant Researcher, Department of Prosthodontics, Christian Albrecht University of Kiel, University Hospital Schleswig-Holstein, Campus Kiel, Arnold-Heller-Strasse 3, 24105 Kiel, Germany. Performed the experiments in partial fulfillment of requirements for a degree, and performed a certain test.; b †Matthias Kern Professor, Department of Prosthodontics, Christian Albrecht University of Kiel, University Hospital Schleswig-Holstein, Campus Kiel, Arnold-Heller-Strasse 3, 24105 Kiel, Germany. Idea, hypothesis. † Deceased.; c Sebastian Wille Assistant Researcher, Department of Prosthodontics, Christian Albrecht University of Kiel, University Hospital Schleswig-Holstein, Campus Kiel, Arnold-Heller-Strasse 3, 24105 Kiel, Germany. Consulted on and performed statistical evaluation, proofread the manuscript.; d Frank Lehmann Adress Titel Firma Anschrift Postdoctoral Researcher, Department of Prosthodontics, Christian Albrecht University of Kiel, University Hospital Schleswig-Holstein, Campus Kiel, Arnold-Heller-Strasse 3, 24105 Kiel, Germany. Consulted on and performed statistical evaluation, proofread the manuscript.; e Sinan Sen Professor and Head, Department of Orthodontics, Christian Albrecht University of Kiel, University Hospital Schleswig-Holstein, Campus Kiel, Arnold-Heller-Strasse 3, 24105 Kiel, Germany. Contributed substantially to the discussion and proofread the manuscript.; f Maximiliane Amelie Schlenz Professor and Head, Department of Prosthodontics, Christian Albrecht University of Kiel, University Hospital Schleswig-Holstein, Campus Kiel, Arnold-Heller-Strasse 3, 24105 Kiel, Germany. Contributed substantially to the discussion and proofread the manuscript.; g Alexander Schmidt Senior Lecturer, Department of Prosthodontics, Christian Albrecht University of Kiel, University Hospital Schleswig-Holstein, Campus Kiel, Arnold-Heller-Strasse 3, 24105 Kiel, Germany. Wrote the manuscript, proofread the manuscript, contributed substantially to discussion.

**Keywords:** aging, air abrasion, dental bonding, orthodontic brackets, shear strength, surface conditioning, thermocycling, zirconia

## Abstract

**Purpose:**

The increasing use of monolithic translucent zirconia for fixed dental restora-tions in orthodontic patients has created new challenges for reliable bracket bonding. This *in vitro* study evaluated the influence of different surface pretreatment protocols on the shear bond strength (SBS) of metal brackets bonded to 3-mol% yttria-stabilized tetragonal zirconia polycrystal (3Y-TZP) and 5-mol% yttria-stabilized partially stabilized zirconia (5Y-PSZ) ceramics using an adhesive resin cement.

**Methods and Materials:**

Ninety-six specimens were prepared (n = 48 per zirconia type), each providing two bonding interfaces (n = 192). Three surface pretreatment protocols were tested: pumice slurry (PS), silicon carbide burr roughening (SiB), and alumina air-particle abrasion (AP). Metal brackets were bonded using an MDP-containing self-adhesive resin cement and a custom 3D-printed bonding device, applying a standardized load of 7.4 N. Half of the specimens were tested for SBS after 3 days of water storage at 37°C; the remaining half after artificial aging (37,500 thermocycles between 5°C and 55°C) for 150 days. SBS was measured using a universal testing machine. Data were analyzed using non-parametric tests (Kruskal–Wallis; Mann–Whitney U with Bonferroni–Holm correction; α = 0.05).

**Results:**

Artificial aging significantly affected SBS, whereas zirconia type had no significant influence. After 3 days, no significant differences were observed among surface pretreatment methods. Following artificial aging, AP and SiB pretreatments achieved significantly higher SBS values than PS for both zirconia types. AP yielded the highest bond strengths overall (up to 51.3 MPa for 5Y-PSZ after 150 days). Spontaneous debonding occurred only in the PS groups.

**Conclusion:**

Although AP pretreatment achieved the highest bond strength values, its clinical application is limited by the risk of surface damage and uncontrollable intraoral pressures. SiB pretreatment provided clinically sufficient bond strength while preserving zirconia surfaces, representing a safer and more practical alternative. Pumice slurry alone was inadequate for reliable bonding. Thus, SiB can be recommended as a minimally invasive and effective clinical protocol for orthodontic bonding to zirconia restorations.

Over the past decades, the demographic profile of orthodontic patients has shifted significantly. While in 1970 only about 5% of orthodontic patients were adults, today this figure has risen to approximately 30%.^5^ Although aligner systems have gained popularity among adult patients – primarily due to esthetic considerations – there remain numerous clinical situations that still necessitate treatment with a conventional multibracket appliance.^36^ Depending on the clinical requirements, these appliances may be placed using lingual, palatal, or vestibular anchorage.^29^ Unlike in younger patients, bonding multibracket appliances in adults is often complicated by the presence of fixed prosthetic restorations. In addition to adult patients, zirconia-based restorations are also increasingly relevant in adolescents. In particular, molar incisor hypomineralization (MIH) represents a frequent developmental condition affecting first permanent molars and incisors, often requiring indirect restorative treatment at a young age.^39^ Reported prevalence rates range between approximately 10% and 20% worldwide.^9^ In cases of severe enamel breakdown, monolithic zirconia restorations may be indicated due to their mechanical stability and reduced minimum thickness requirements. Consequently, orthodontic bracket bonding to zirconia restorations may also become clinically relevant in adolescent patients undergoing fixed appliance therapy.

Epidemiological data indicate that indirect restorations are common in adult populations and increase with age. National survey data, such as the Sixth German Oral Health Study (DMS VI), demonstrate that a considerable proportion of middle-aged adults present with crowns or implant-supported restorations.^40^ These findings are consistent with international evidence. In a comprehensive systematic review, Pjetursson et al^30^ reported high survival rates and widespread use of fixed prosthetic restorations in adults over 40 years of age. Likewise, global burden analyses have shown a substantial prevalence of untreated caries and tooth loss worldwide, which represent major drivers for indirect restorative treatment.^16^ Together, these data underline that orthodontic treatment in adults frequently involves bonding procedures on restored teeth rather than intact enamel.

Comparable results were observed in a Finnish population study, indicating that nearly 12% of older adults were restored with single crowns.^27^ In addition, there are adolescent patients who receive zirconia fixed dental prostheses (FDPs) due to dental trauma or congenital tooth agenesis, such as cantilever zirconia resin-bonded fixed dental prostheses (RBFDPs) in the anterior or posterior region, replacing missing teeth.^2,25^

All-ceramic materials have become increasingly important in the context of single crown restorations, as they offer a combination of high mechanical strength and superior esthetics.^35^ Compared to lithium disilicate, monolithic zirconia is being used more frequently, primarily due to its reduced susceptibility to chipping fractures and its lower minimum layer thickness requirements, which allow for a more tissue-preserving preparation.^3^ However, one significant limitation of zirconia is its reduced bonding capability compared to other restorative materials.^1^

These restorative situations pose significant challenges for bracket adhesion, as conventional adhesive techniques developed for enamel are not directly applicable. Consequently, the incidence of debonding is higher when orthodontic brackets are bonded to zirconia restorations compared to enamel.^26^ The standard procedure for bracket placement on enamel involves acid etching, the application of a bonding agent, and subsequent attachment using a low-viscosity composite material, allowing for reversible bonding.

However, this approach would result in insufficient bracket adhesion on ceramic restorations. Furthermore, in clinical practice, it is often difficult to determine the exact type of ceramic material involved, as different ceramics are visually difficult to distinguish from one another.^18^

This poses a problem, as different ceramic materials – such as zirconia and lithium disilicate –require different preconditioning protocols.^7,32^ For example, silica-based ceramics require etching with hydrofluoric acid followed by the application of a silane coupling agent, whereas metal oxide ceramics, such as zirconia, necessitate air-particle abrasion^41^ and subsequent treatment with a primer containing adhesive phosphate monomers.^4^ Therefore, failure to correctly identify the ceramic material and apply the corresponding pretreatment protocol may lead to suboptimal bond strength and clinical failure.^28^

In addition to these material-specific challenges, two clinically relevant considerations must be taken into account when bonding orthodontic brackets to zirconia restorations. First, a clinically acceptable level of shear bond strength (SBS) is necessary to ensure uninterrupted orthodontic treatment, as repeated debonding and rebonding procedures are undesirable for both clinicians and patients. Second, excessively high SBS values carry the risk of inducing cracks or fractures in the ceramic substrate during debonding, which may necessitate additional prosthodontic intervention. These opposing requirements highlight the importance of achieving a balance between adequate bracket retention during treatment and predictable, damage-free debonding at its conclusion.

Given the associated challenges in clinical practice, it is desirable to establish a universally applicable protocol for the bonding of orthodontic brackets to zirconia ceramics. Ideally, such a protocol should provide SBS within an optimal range – sufficiently high to prevent premature bracket failure during orthodontic treatment, yet controlled enough to allow predictable and damage-free debonding at the end of therapy. This is particularly relevant as, in response to the growing demand for higher translucency, newer generations of zirconia, especially monolithic restorations, are increasingly used. These developments highlight the need for reliable and simplified bonding strategies, particularly in the context of orthodontic treatment involving zirconia-based restorations. Since zirconia represents a non-etchable, oxide-based ceramic, the selection of a suitable adhesive system is crucial. In the present study, a self-adhesive resin cement containing the functional monomer 10-MDP was chosen, as this monomer is known to chemically interact with zirconium oxide.^32,41^ The intention was not to replicate conventional enamel-bonding protocols used in orthodontics, but rather to investigate a material-oriented bonding strategy that specifically addresses the zirconia substrate. Additionally, the use of a self-adhesive system reflects a clinically simplified approach that may facilitate chairside application in interdisciplinary treatment settings.

Therefore, the aim of this study was to investigate whether the shear bond strength (SBS) of metal brackets to 3 mol% yttria-stabilized tetragonal zirconia polycrystal (3Y-TZP) and 5 mol% yttria partially stabilized zirconia (5Y-PSZ) ceramics is influenced by the type of surface pretreatment when using an adhesive cement. This could serve as the basis for simplifying the bonding procedure at the interface between orthodontics and dental prosthetics.

The following hypothesis was tested: The type of surface pretreatment has no effect on the SBS of metal brackets bonded with adhesive cement to different types of zirconia.

## METHODS AND MATERIALS

### Specimen Preparation

Two groups of zirconia types with distinct compositions were investigated. In total, 96 specimens were prepared, with 48 specimens assigned to each zirconia type. Since each specimen provided two bonding interfaces, this resulted in a total of 192 bonding surfaces. For each zirconia type, 16 bonding interfaces were allocated to each of the three surface pretreatment protocols (n = 16), which were subsequently tested under two conditions (non-aged and aged). Accordingly, 96 bonding interfaces were evaluated per zirconia type.

The first group comprised specimens fabricated from 3 mol% yttria-stabilized tetragonal zirconia polycrystal (3Y-TZP, KATANA Zirconia HT, Kuraray Noritake, Tokyo, Japan). The second group consisted of specimens made from 5 mol% yttria partially stabilized zirconia (5Y-PSZ, KATANA Zirconia UTML, Kuraray Noritake, Tokyo, Japan).

**Fig 1:**
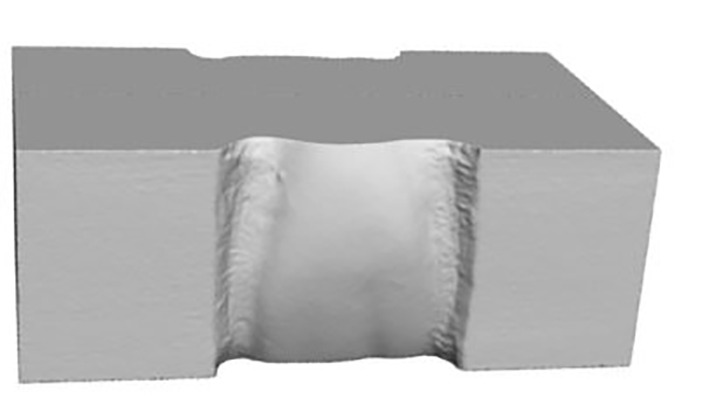
Three-dimensional rendering generated from the standard tessellation language (STL) file used for the milling of the ceramic test specimens. The front and back surfaces replicate the vestibular contour of a maxillary right central incisor and serve as the designated bonding areas.

Each group consisted of cuboid-shaped test specimens. On two opposing surfaces of each specimen, the vestibular contour of a maxillary right central incisor was milled to simulate the clinical bonding interface. These milled surfaces served as the designated bonding areas (Fig 1).

Each of the two main zirconia groups (3Y-TZP and 5Y-PSZ) was randomly divided into three subgroups, each subjected to a different surface pretreatment protocol:

**Group PS (pumice slurry):** After milling and sintering, the bonding surfaces were cleaned using a pumice slurry (Bimsmehl, Ernst-Hinrichs Dental, Goslar, Germany).
**Group SiB (silicon carbide burr):** Surface roughening was performed using a silicon carbide burr (SiC-Schleifer, VOCO, Offenbach, Germany).
**Group AP (alumina particles):** The surfaces were treated by air-particle abrasion with 50 μm alumina particles (Edelkorund Weiß, 99.8% Al_2_O_3_, Pluradent, Offenbach, Germany) using an airborne-particle abrasion unit (P-G400; Harnisch + Rieth, Winterbach, Germany) at one bar pressure.

### Bracket Bonding

**Fig 2a to i:**
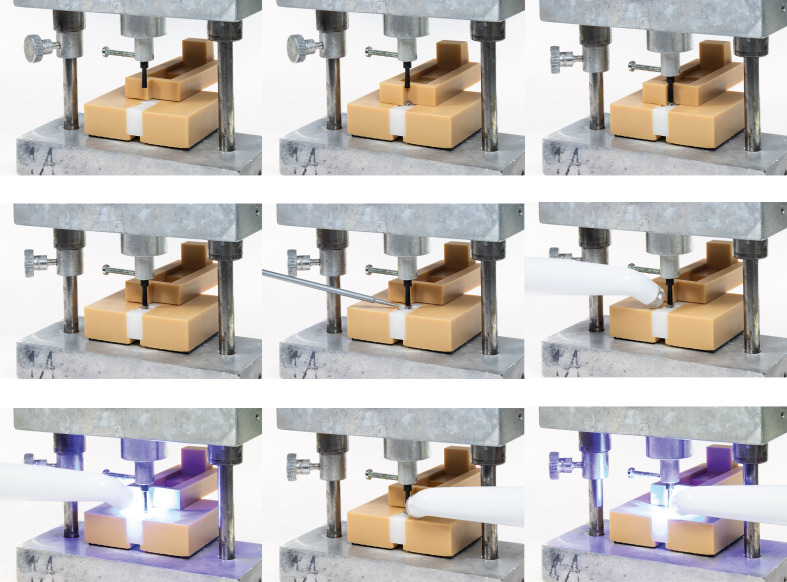
Schematic illustration of the bonding device to ensure standardized bracket placement and adhesion. (a) The test specimen is placed into the bonding device while the stamp is raised and the guiding part is moved backward. (b) The bracket is positioned in the predefined corner of the guiding part. (c) The stamp is lowered to apply a standardized force of 750 g, pressing the bracket onto the bonding surface. (d) The guiding part is moved backward while the stamp holds the bracket in position. (e) Marginally extruded cement is carefully removed using a microbrush, without applying pressure to the bracket body. (f to i) The cement is light-cured, securing the bracket to the test specimen.

Subsequently, a metal bracket for the maxillary right central incisor (Sprint®-II, Forestadent, Pforzheim, Germany) was bonded using a self-adhesive resin cement containing 10-Methacryloyloxydecyl dihydrogen phosphate (MDP; PANAVIA SA Cement Universal; Kuraray Noritake, Tokyo, Japan). The bracket was positioned using a self-designed and 3D-printed bonding device, which enabled standardized placement of the brackets onto the differently pretreated bonding surfaces (PS, AP, and SiB). The device applied a defined load corresponding to the weight of the sliding carriage (750 g), resulting in a constant bonding pressure of approximately 7.4 N. After bracket positioning and stabilization using the positioning device, excess cement was removed immediately and prior to light polymerization. Once the positioning guide was retracted, marginally extruded cement became accessible from all sides and was carefully eliminated using a microbrush (Microbrush in grey, Pluradent, Offenbach, Germany). Only the cement that had flowed beyond the bracket margins was removed. The procedure was performed using gentle pulling strokes without applying pressure to the bracket body in order to avoid displacement. This approach ensured that the cement layer thickness beneath the bracket base remained unaffected. All cement removal procedures were performed by a single operator to ensure standardized handling conditions. The adhesive was then light-cured with a polymerization unit (Radii-Cal, SDI, Victoria, Australia). The procedure is illustrated in Figure 2.

### Artificial Aging and Shear Bond Strength Testing

After 3 days of water storage at 37°C (B-Tron-Hydroprocessor, Pentron, Orange, USA) a randomly selected half of each subgroup was mounted in a universal testing machine (Zwick Z010, Zwick & Co, Ulm, Germany), and the bracket was debonded using a shear force applied at a crosshead speed of 2 mm/min to determine the SBS.

The remaining half of each subgroup underwent an aging protocol lasting 150 days, alternating between thermocycling (Thermocycler, Willytec, Munich, Germany) and water storage at 37°C. A total of 37,500 thermocycles were performed between 5°C and 55°C. After aging, these specimens were also mounted in the Zwick universal testing machine, and the brackets were tested using a shear force, as described by Fischer-Brandies et al.^11^ The SBS of the differently conditioned substrates was recorded in Newtons using the testXpert II software (ZwickRoell, Zwick, Ulm, Germany).

### Failure Mode Analysis (ARI)

After shear bond strength testing, all specimens were subjected to failure mode analysis using the Adhesive Remnant Index (ARI). Debonded zirconia surfaces and bracket bases were examined under a stereomicroscope at 25× magnification (Stereomicroscope Zeiss S7, Carl Zeiss, Oberkochen, Germany). ARI scores were assigned as follows (adapted to zirconia surfaces):

ARI 0 = no cement remaining on the zirconia surface;
ARI 1 = less than 50% cement remaining;
ARI 2 = more than 50% cement remaining;
ARI 3 = all cement remaining on the zirconia surface.

All evaluations were performed by a single examiner.

### Scanning Electron Microscopy (SEM)

Representative zirconia surfaces and fracture interfaces were examined using scanning electron microscopy (SEM) (ZEISS Gemini Ultra 55 Plus, Carl Zeiss, Oberkochen, Germany) at an accelerating voltage of 2 kV.

Specimens were mounted on aluminum stubs using conductive carbon tape and sputter-coated with gold (VAC COAT DSCR, VAC COAT Ltd., UK) at 10 mA for 300 s in auto mode.

**Fig 3:**
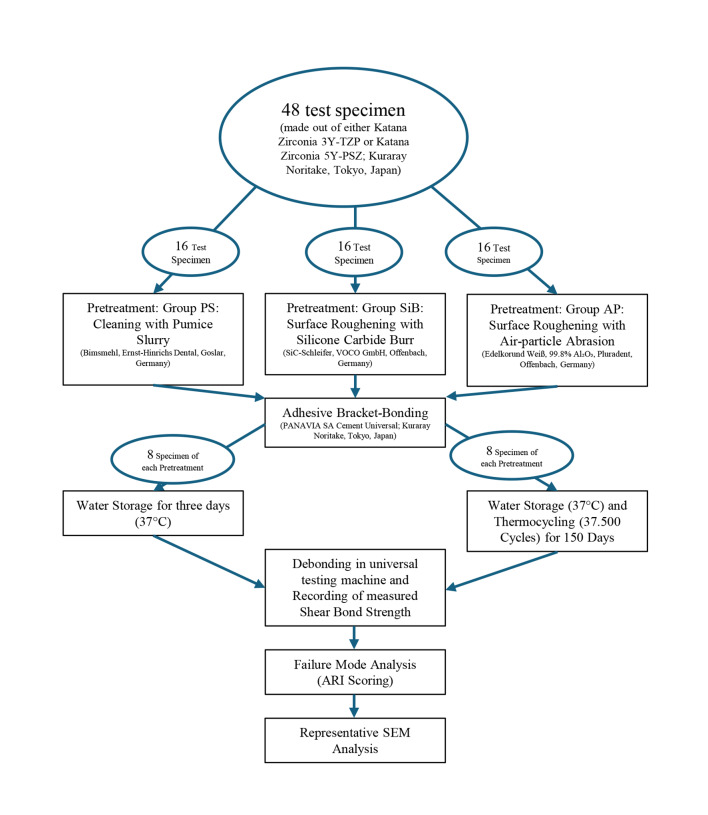
Flowchart illustrating the experimental workflow for each zirconia type (3Y-TZP and 5Y-PSZ). Each test specimen provided two independent bonding surfaces, resulting in 96 bonding interfaces per zirconia type (n = 192 in total). PS = pumice cleaning; SiB = silicon carbide burr roughening; AP = air-particle abrasion; ARI = Adhesive Remnant Index; SEM = scanning electron microscopy.

Micrographs were recorded at magnifications ranging from approximately 230× to 650× for qualitative evaluation of surface morphology and fracture characteristics. For better clarity, the overall study procedure is illustrated in Figure 3.

Statistical analysis was performed using Microsoft Excel and IBM SPSS Statistics version 29. The Shapiro–Wilk test revealed a non-normal distribution of the data (*P* ≤ 0.05); therefore, the non-parametric Kruskal–Wallis test was applied. Pairwise comparisons between groups based on material, aging duration, and surface pretreatment were conducted using the Mann–Whitney U test, with Bonferroni–Holm correction to adjust for multiple comparisons.

To allow comparison of the measured SBS values (in Newtons) with those reported in the literature (in MPa),^31^ the forces were normalized by dividing them by the adhesive surface area of the bracket, which is 10.8 mm^2^.

## RESULTS

**Fig 4:**
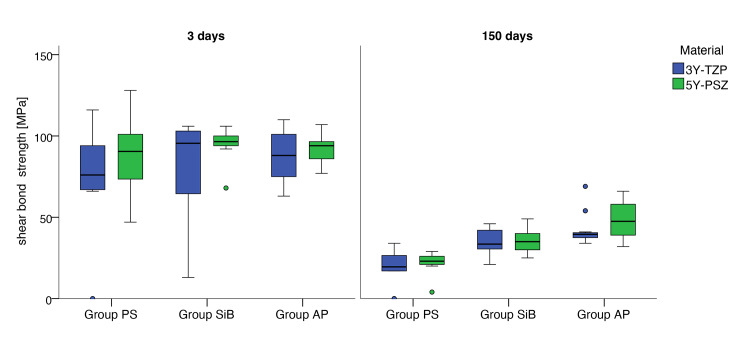
Boxplots showing the influence of storage time and surface pretreatment on shear bond strength (SBS, in MPa) for 3Y-TZP and 5Y-PSZ zirconia.

Table 1 and Figure 4 present the evaluation of the datasets by storage duration, zirconia type, and surface pretreatment.

**Table 1:** Shear bond strength (SBS) values in Newtons (N), expressed as mean (standard deviation) and quartiles (Q25, Q50, Q75).

Storage time [d]	Zirconia type/group	Surface pretreatment	N	x̄	σ	Q25	Q50	Q75
3 d	3Y-TZP	PS	16	84.1	28.3	72.7	82.3	100.8
		SiB	16	89.2	28.0	70.0	103.0	111.0
		AP	16	94.0	16.6	82.2	94.8	108.5
	5Y-PSZ	PS	16	94.1	21.6	80.2	97.7	108.0
		SiB	16	103.3	8.8	101.8	104.5	108.0
		AP	16	99.2	8.4	92.5	101.0	104.3
150 d	3Y-TZP	PS	16	20.9	9.8	18.7	21.3 B	28.6
		SiB	16	37.7	7.7	33.4	36.5 A	45.2
		AP	16	45.6	9.9	41.3	42.6 A	43.7
	5Y-PSZ	PS	16	24.2	6.7	22.3	25.1 C	28.2
		SiB	16	38.3	7.5	32.8	38.2 B	42.4
		AP	16	51.3	11.8	42.7	51.2 A	61.8
Statistically significant differences (*P* < 0.05) are indicated by different uppercase letters within each material and storage time. The table includes the number of specimens (N), the mean (x̄), the standard deviation (σ), and the 25th, 50th, and 75th percentiles, with Q50 representing the median								

In the PS group, spontaneous debonding of the adhesive interface occurred in one specimen after 3 days and in two specimens after 150 days. These were included in the statistical analysis with an SBS of 0 MPa. No spontaneous debonding was observed in any other subgroup.

The Shapiro–Wilk test indicated that storage duration and artificial aging had a significant effect on SBS in all groups, whereas the base material had no significant influence. After 3 days of storage, no significant differences were found between any of the pretreatment options.

After 150 days of storage in the thermal cycler, comparison of the pretreatment methods revealed that, within the 3Y-TZP group, the SiB group did not differ significantly from the AP group after Bonferroni–Holm correction, whereas both groups showed significantly higher values than the PS group. In the 5Y-PSZ group, all pretreatment methods differed significantly from each other.

### Failure Mode Analysis (ARI)

Failure modes after debonding were assessed using the ARI, and the distribution of scores is presented in Table 2.

**Table 2:** Distribution of Adhesive Remnant Index (ARI) scores (n = 16 per group) according to zirconia type, surface pretreatment, and aging condition (3 days or 150 days).

Storage time [d]	Zirconia type/group	Surface pretreatment	N	ARI 0	ARI 1	ARI 2	ARI 3
3 d	3Y-TZP	PS	16	0	5	9	2
		SiB	16	0	1	8	7
		AP	16	0	0	13	3
	5Y-PSZ	PS	16	0	2	13	1
		SiB	16	0	0	4	12
		AP	16	0	0	7	9
150 d	3Y-TZP	PS	16	16	0	0	0
		SiB	16	15	1	0	0
		AP	16	15	1	0	0
	5Y-PSZ	PS	16	15	1	0	0
		SiB	16	12	4	0	0
		AP	16	10	6	0	0
PS = pumice cleaning; SiB = silicon carbide burr roughening; AP = air-particle abrasion							

After 3 days of water storage, most specimens across all pretreatment groups exhibited ARI scores 2 and 3, indicating substantial cement retention on the zirconia surface. In the PS groups, ARI 1 and 2 were predominant, whereas in the SiB and AP groups, higher frequencies of ARI 2 and 3 were observed. In particular, SiB (5Y-PSZ) and AP (5Y-PSZ) showed a high proportion of ARI 3 scores after 3 days.

After artificial aging (150 days, 37,500 thermocycles), the ARI distribution shifted markedly toward lower scores. In all pretreatment groups and both zirconia types, ARI 0 predominated, indicating predominantly adhesive failure at the zirconia–cement interface. ARI 2 and 3 were no longer observed after aging, and only isolated ARI 1 scores occurred in some groups.

### Representative SEM Micrographs

**Fig 5a to f:**
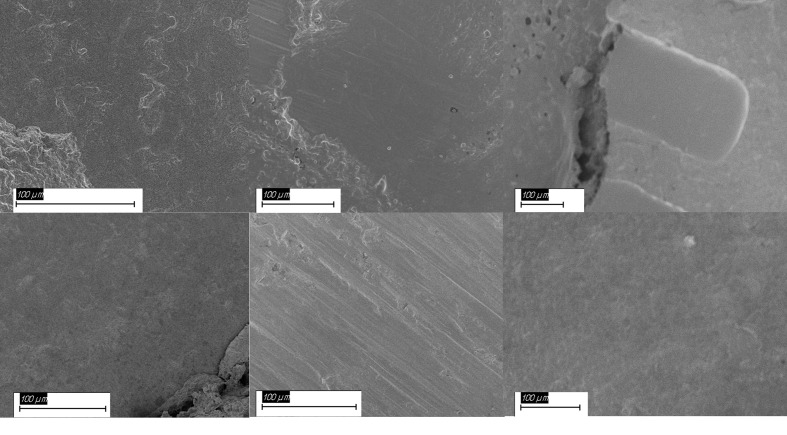
Representative scanning electron micrographs (SEM) of zirconia surfaces and fracture patterns. (a to c) Surface morphology of 3Y-TZP after pretreatment and 3 days of water storage: (a) PS (~635×), (b) SiB (~390×), (c) AP (~231×). (d to f) Fracture surfaces after artificial aging (37,500 thermocycles): (d) PS (~468×), (e) SiB (~511×), (f) AP (~321×). Scale bars: 100 µm.

Representative SEM micrographs are shown in Figure 5. Clear differences in surface morphology were observed between the pretreatment protocols after 3 days of water storage (Fig 5a to c). PS-treated specimens exhibited relatively smooth surfaces with minor superficial irregularities. SiB pretreatment resulted in linear grooves and moderate roughness, whereas AP produced a more irregular and micro-retentive surface topography.

Fracture surface analysis after artificial aging (37,500 thermocycles) revealed predominantly interfacial failure patterns (Fig 5d to f). Most aged specimens showed largely cement-free zirconia surfaces, consistent with the predominance of ARI 0. Occasional marginal cement remnants were located outside the effective bonding interface.

## DISCUSSION

The null hypothesis that the type of surface pretreatment has no effect on SBS had to be rejected, as the results of this study clearly demonstrate that different pretreatment methods significantly influence the SBS of metal brackets to 3Y-TZP and 5Y-PSZ zirconia when using adhesive cement. It should be emphasized that the adhesive system used in this study was deliberately selected based on its chemical affinity to zirconium oxide rather than on conventional orthodontic enamel-bonding standards. The presence of 10-MDP allows for chemical interaction with zirconia, which is considered essential for durable bonding to oxide ceramics. Therefore, the present investigation was designed from a substrate-oriented perspective, focusing on the zirconia–cement interface rather than on traditional orthodontic adhesive protocols.

Both alumina air-particle abrasion (AP) and the surface roughening with a silicon carbide burr (SiB) proved beneficial for achieving reliable bond strength. In contrast, pretreatment with pumice slurry (PS) did not provide sufficient surface roughening for durable adhesion, a finding consistent with the results of Kwak et al.^19^ Nevertheless, it could still be useful as a cleaning step for pretreated zirconia prior to bonding.^6,10^

When directly comparing AP and SiB, AP achieved slightly higher SBS values. Similar observations were made by Lee et al,^21^ who reported clinically sufficient values after air abrasion combined with different zirconia adhesives or a universal adhesive.

Likewise, Libecki et al^22^ confirmed the superiority of air abrasion over silane application, although they also found SiB to result in adequate, albeit slightly lower, bond strengths.

However, the literature provides inconsistent evidence regarding the relative effectiveness of air abrasion and silane treatment. While Gomes et al^12^ and Lee et al^20^ describe sufficient adhesion after both approaches, Mokhtarpur et al^24^ report contradictory results.

Despite the somewhat higher values achieved with AP in the present study, there are strong clinical and material-science arguments for favoring SiB pretreatment. First, SiB is less invasive: alumina particle abrasion has been shown to significantly reduce the flexural strength of 5Y-PSZ zirconia, potentially compromising the integrity of crowns after orthodontic treatment, as shown by Hergeröder et al.^14^

Second, the clinical application of AP is problematic. Since FDPs cannot be removed intraorally for controlled surface preparation, blasting must be performed with chairside devices. These emit turbine-dependent pressures that cannot be regulated and often exceed one bar which, as demonstrated by Kern et al,^17^ can damage zirconia surfaces regardless of whether it is 3Y-TZP or 5Y-PSZ.

When interpreting these findings, the clinical context and treatment objectives must also be considered. Orthodontic bonding differs fundamentally from prosthetic cementation. Orthodontic attachments are designed for semi-permanent fixation: treatment typically lasts less than 2 years,^38^ and the bond must be strong enough to prevent debonding during therapy, yet weak enough to allow bracket removal without damaging the underlying surface.^8^ An additional clinical reference point is the frequently cited minimum shear bond strength threshold of 5.9–7.8 MPa, originally proposed by Reynolds,^31^ and still referenced in recent literature.^34^ SBS values above this range are generally considered sufficient to avoid unintended bracket loss. However, when bonding to zirconia restorations, these thresholds must be interpreted with caution. While inadequate bond strength increases the risk of premature failure, excessively high adhesion may jeopardize the integrity of the ceramic substrate during debonding. Thus, the clinical objective is to achieve a balance between reliable retention throughout treatment and safe, predictable bracket removal without damaging the underlying restoration.

In this context, the findings of Jungbauer et al^15^ are noteworthy: across different pretreatment protocols, adhesives, and aging regimens, SBS values ranged from 8.3 to 16.9 MPa, with all tested combinations meeting clinically acceptable thresholds. Their results further underline that zirconia–bracket interfaces can achieve adequate retention while still remaining within a clinically manageable debonding range.

Consequently, the maximum SBS should remain below 13 MPa to minimize the risk of fractures, as suggested by Thurmond et al.^37^ Within this framework, bond failures are generally less problematic than damage to the crown or tooth substrate. This distinction also illustrates why lower SBS in orthodontics is not necessarily disadvantageous compared to prosthetics. In fact, lower values can be beneficial, as they facilitate safe bracket removal without compromising the underlying zirconia restoration. For instance, Hansen et al^13^ observed significantly higher values under otherwise comparable conditions, a difference attributable to the intended permanence of prosthetic cementation.

An additional aspect that must be considered when interpreting the present results is the role of artificial aging through thermocycling. The number of cycles applied has a direct influence on the measured SBS values. Several previous investigations on the bonding of brackets to zirconia used significantly shorter thermocycling protocols, which explains the higher bond strengths they reported. For example, Kwak et al^19^ applied only 1000 cycles, Heidari et al (33) used 2000 cycles, and Mehmeti et al^23^ performed 5800 cycles. In all these studies, the resulting SBS were noticeably higher than those observed in the present investigation. Similarly, Jungbauer et al^15^ evaluated SBS after 24 h, 500 thermocycles, and up to 90 days of storage at 37°C and reported SBS values between 8.3 and 16.9 MPa, depending on pretreatment and adhesive system. Their data confirm that shorter aging protocols or lower thermocycling loads tend to preserve higher SBS values, whereas extended aging – as applied in the present study – more clearly reveals long-term degradation at the bracket–zirconia interface.

In contrast, the current study deliberately adopted 37,500 cycles, as commonly employed in prosthodontic research, to allow better comparability with data from previous studies.

This extended aging protocol is clinically meaningful, since it simulates long-term intraoral exposure with repeated thermal fluctuations and moisture. However, it must be acknowledged that such prolonged thermocycling places greater hydrolytic and thermal stress on the resin components of the bonding system, ultimately leading to reduced adhesion values. Thus, while shorter cycling protocols such as those of Kwak, Heidari, and Mehmeti provide insights into early-phase bond stability, the present study indicates how zirconia–bracket interfaces may behave under more demanding, long-term conditions. This discrepancy in methodology highlights the importance of carefully considering thermocycling duration when comparing results across different studies.

The observed shift in failure mode distribution after artificial aging provides further insight into the degradation mechanisms at the zirconia–cement interface. After 3 days, higher ARI scores (2–3) indicated substantial cement retention on the zirconia surface, suggesting that failure frequently occurred within the resin cement or at the bracket–cement interface. This finding is consistent with the SEM observations showing pronounced surface roughness after SiB and AP pretreatment, which likely contributed to enhanced micromechanical interlocking.

Following 37,500 thermocycles, the failure pattern shifted predominantly toward ARI 0 across all pretreatment groups. SEM analysis confirmed that most aged specimens exhibited largely cement-free zirconia surfaces, indicating failure primarily at the zirconia–cement interface. This shift suggests that long-term hydrothermal stress may weaken the interfacial bond, even in the presence of micromechanical retention. The reduction in cohesive cement remnants after aging reflects a degradation of the adhesive interface rather than changes in the substrate morphology itself.

These findings underline the importance of evaluating bonding protocols under extended aging conditions. While mechanical surface pretreatment enhances initial bond strength, long-term durability appears to be governed by the stability of the chemical interaction between the adhesive system and zirconium oxide.

In light of both the quantitative bond strength results and the observed fracture patterns, although AP pretreatment yielded slightly higher SBS, SiB application should be preferred in clinical orthodontics due to its protective effect on zirconia ceramics and its reduced risk of surface damage.

## CONCLUSIONS

Within the limitations of this *in vitro* study, it can be concluded that both alumina AP and the surface roughening with a silicon carbide burr (SiB) enable clinically sufficient bonding of metal brackets to 3Y-TZP and 5Y-PSZ zirconia ceramics. Although AP pretreatment resulted in the highest SBS, its clinical use remains questionable: alumina abrasion may damage the ceramic surface and, due to the uncontrollable pressure of intraoral devices, cannot be reliably applied in practice.

SiB pretreatment, by contrast, represents a clinically viable and safer option. It ensures sufficient adhesion while preserving the structural integrity of zirconia, particularly 5Y-PSZ. The use of SiB for pretreatment also does not threaten the integrity of zirconia, but its somewhat lower bond values may result in occasional bracket reattachment during orthodontic therapy. Pumice slurry, on the other hand, did not provide adequate roughening and cannot be recommended as a pretreatment method.

Considering the semi-permanent character of orthodontic bonding, SBS do not need to reach prosthetic levels. Rather, adhesion must be strong enough to retain brackets during treatment, but weak enough to allow safe removal afterward without compromising the restoration. The present results confirm that this balance can best be achieved with SiB pretreatment, offering a reliable, less invasive, and clinically practical protocol for bonding orthodontic appliances to zirconia-based crowns.

### Acknowledgments

The authors would like to thank Sebastian Ahsbahs for providing the brackets. The authors gratefully acknowledge MSc Barnika Chakraborty for technical support with SEM imaging. The authors also thank Prof Dr Rainer Adelung (Functional Nanomaterials, Institute for Materials Science, Faculty of Engineering, Christian-Albrechts-University Kiel) for providing access to the microscopy facilities.

#### Clinical relevance

Reliable bonding of orthodontic brackets to zirconia restorations is increasingly important as both the number of adult orthodontic patients and the prevalence of monolithic zirconia crowns continue to rise. Clinicians require pretreatment protocols that provide sufficient retention during treatment while minimizing the risk of ceramic damage during debonding. The present findings support silicon carbide burr roughening as a clinically practical and minimally invasive approach to achieving predictable bracket adhesion on zirconia surfaces.

## References

[ref1] Ahmed T, Fareen N, Alam MK (2021). The effect of surface treatment and thermocycling on the shear bond strength of orthodontic brackets to the Y-TZP zirconia ceramics: a systematic review. Dental Press J Orthod.

[ref2] Alghauli MA, Wille S, Lehmann F, Kern M (2023). Survival and debonding resistance of posterior cantilever resin-bonded fixed dental prostheses for moderately and severely worn dentition during thermomechanical loading. Dent Mater.

[ref3] Bacchi A, Cesar PF (2022). Advances in ceramics for dental applications. Dent Clin North Am.

[ref4] Blatz MB, Conejo J, Alammar A, Ayub J (2022). Current protocols for resin-bonded dental ceramics. Dent Clin North Am.

[ref5] Christensen L, Luther F (2015). Adults seeking orthodontic treatment: expectations, periodontal and TMD issues. Br Dent J.

[ref6] Demir N, Genc O, Akkese IB, Malkoc MA, Ozcan M (2024). Bonding effectiveness of saliva-contaminated monolithic zirconia ceramics using different decontamination protocols. Biomed Res Int.

[ref7] Dos Santos D, Pacheco RR, Komegae GH, da Silva JA, Lima D, Pini N (2023). Effects of hydrofluoric acid concentrations, commercial brands, and adhesive application on the bond strength of a resin luting agent to lithium disilicate glass ceramic. Oper Dent.

[ref8] Dumbryte I, Vebriene J, Linkeviciene L, Malinauskas M (2018). Enamel microcracks in the form of tooth damage during orthodontic debonding: a systematic review and meta-analysis of in vitro studies. Eur J Orthod.

[ref9] Elhennawy K, Schwendicke F (2016). Managing molar-incisor hypomineralization: a systematic review. J Dent.

[ref10] Fichtel T, Crha M, Langerová E, Biberauer G, Vlašín M (2008). Observations on the effects of scaling and polishing methods on enamel. J Vet Dent.

[ref11] Fischer-Brandies H, Kremers L, Reicheneder C, Kluge G, Husler K (1993). Enamel damage depending on the method of bracket removal. Fortschr Kieferorthop.

[ref12] Gomes AL, Ramos JC, Santos-del Riego S, Montero J, Albaladejo A (2015). Thermocycling effect on microshear bond strength to zirconia ceramic using Er:YAG and tribochemical silica coating as surface conditioning. Lasers Med Sci.

[ref13] Hansen NA, Wille S, Kern M (2020). Effect of reduced airborne-particle abrasion pressure on the retention of zirconia copings resin bonded to titanium abutments. J Prosthet Dent.

[ref14] Hergeröder C, Wille S, Kern M (2022). Comparison of testing designs for flexural strength of 3Y-TZP and 5Y-PSZ considering different surface treatment. Materials (Basel).

[ref15] Jungbauer R, Proff P, Edelhoff D, Stawarczyk B (2022). Impact of different pretreatments and attachment materials on shear bond strength between monolithic zirconia restorations and metal brackets. Sci Rep.

[ref16] Kassebaum NJ, Bernabe E, Dahiya M, Bhandari B, Murray CJ, Marcenes W (2015). Global burden of untreated caries: a systematic review and metaregression. J Dent Res.

[ref17] Kern M, Barloi A, Yang B (2009). Surface conditioning influences zirconia ceramic bonding. J Dent Res.

[ref18] Kontonasaki E, Rigos AE, Ilia C, Istantsos T (2019). Monolithic zirconia: an update to current knowledge. Optical properties, wear, and clinical performance. Dent J (Basel).

[ref19] Kwak JY, Jung HK, Choi IK, Kwon TY (2016). Orthodontic bracket bonding to glazed full-contour zirconia. Restor Dent Endod.

[ref20] Lee JH, Lee M, Kim KN, Hwang CJ (2015). Resin bonding of metal brackets to glazed zirconia with a porcelain primer. Korean J Orthod.

[ref21] Lee JY, Ahn J, An SI, Park JW (2018). Comparison of bond strengths of ceramic brackets bonded to zirconia surfaces using different zirconia primers and a universal adhesive. Restor Dent Endod.

[ref22] Libecki W, Elsayed A, Lehmann F, Kern M (2017). Efficacy of different surface treatments for intraoral repair of veneered zirconia frameworks. J Adhes Dent.

[ref23] Mehmeti B, Kelmendi J, Iiljazi-Shahiqi D, Azizi B, Jakovljevic S, Haliti F (2019). Comparison of shear bond strength orthodontic brackets bonded to zirconia and lithium disilicate crowns. Acta Stomatol Croat.

[ref24] Mokhtarpur H, Nafisifard M, Dadgar S, Etemadi A, Chiniforush N, Sobouti F (2020). Shear bond strength of the metal bracket to zirconium ceramic restoration treated by the Nd:YAG laser and other methods: an in vitro microscopic study. J Lasers Med Sci.

[ref25] Mourshed B, Samran A, Alfagih A, Samran A, Abdulrab S, Kern M (2018). Anterior cantilever resin-bonded fixed dental prostheses: a review of the literature. J Prosthodont.

[ref26] Namvar F, Shahrebabak MZ, Arab S, Tabrizi MA, Ghasemi A, Safari F (2022). Shear bond strength of metallic orthodontic brackets to zirconium crowns treated by sandblasting compared to enamel using a universal adhesive: an in vitro study. Int Orthod.

[ref27] Näpänkangas R, Haikola B, Oikarinen K, Söderholm AL, Remes-Lyly T, Sipilä K (2011). Prevalence of single crowns and fixed partial dentures in elderly citizens in the southern and northern parts of Finland. J Oral Rehabil.

[ref29] Patano A, Inchingolo AD, Malcangi G, Garibaldi M, De Leonardis N, Campanelli M (2023). Direct and indirect bonding techniques in orthodontics: a systematic review. Eur Rev Med Pharmacol Sci.

[ref31] Reynolds IR, von Fraunhofer JA (1976). Direct bonding of orthodontic brackets — a comparative study of adhesives. Br J Orthod.

[ref33] Shamohammadi Heidari M, Moradinejad M, Tabatabaei H, Rakhshan V (2022). Effects of three novel bracket luting agents containing zirconia primer on shear bond strength of metal orthodontic brackets attached to monolithic zirconia crowns: a preliminary in vitro study. Int J Dent.

[ref34] Sharma S, Tandon P, Nagar A, Singh GP, Singh A, Chugh VK (2014). A comparison of shear bond strength of orthodontic brackets bonded with four different orthodontic adhesives. J Orthod Sci.

[ref35] Silva LHD, Lima E, Miranda RBP, Favero SS, Lohbauer U, Cesar PF (2017). Dental ceramics: a review of new materials and processing methods. Braz Oral Res.

[ref36] Singla S, Kamboj M, Gupta P, Lehl G, Talwar M (2022). Clinical evaluation of periodontal status in subjects with multibracket appliances and the role of age and gender during initial months of fixed orthodontic treatment. J Indian Soc Periodontol.

[ref37] Thurmond JW, Barkmeier WW, Wilwerding TM (1994). Effect of porcelain surface treatments on bond strengths of composite resin bonded to porcelain. J Prosthet Dent.

[ref38] Tsichlaki A, Chin SY, Pandis N, Fleming PS (2016). How long does treatment with fixed orthodontic appliances last? A systematic review. Am J Orthod Dentofacial Orthop.

[ref39] Weerheijm KL, Jalevik B, Alaluusua S (2001). Molar-incisor hypomineralisation. Caries Res.

[ref40] Wöstmann B, Samietz S, Jordan AR, Kuhr K, Nitschke I, Stark H (2025). Tooth loss and denture status: results of the 6th German Oral Health Study (DMS•6). Quintessence Int.

[ref41] Yang B, Barloi A, Kern M (2010). Influence of air-abrasion on zirconia ceramic bonding using an adhesive composite resin. Dent Mater.

